# Treatment results for patients with squamous-cell carcinoma of the anus, a single institution retrospective analysis

**DOI:** 10.1186/s13014-022-02049-8

**Published:** 2022-04-20

**Authors:** Karen J. Neelis, Django M. Kip, Frank M. Speetjens, Yvette M. van der Linden

**Affiliations:** 1 Department of Radiotherapy, Leiden University Medical Center, Albinusdreef 2, 2333 ZA Leiden, The Netherlands; 2 Department of Medical Oncology, Leiden University Medical Center, Leiden, The Netherlands; 3 Netherlands Comprehensive Cancer Organisation (IKNL), Utrecht, The Netherlands

**Keywords:** Anal carcinoma, Retrospective, Chemoradiotherapy, Treatment results

## Abstract

**Background and purpose:**

To gain insight into the treatment outcomes for anal cancer a retrospective analysis was performed with a special emphasis on trends in outcome and toxicities over time and on treatment of elderly patients.

**Materials and methods:**

Medical records of 98 consecutive patients with squamous cell carcinoma of the anus of all stages treated with curative intent between 01-01-2009 and 31-12-2018 were analyzed with follow up until 31-12-2020. Standard tumor and pathological lymph node dose were 59.4 Gy (median 59.4 Gy, range 59.4–70 Gy) or 60 Gy (no deviation from intended dose), elective nodal regions were treated with 45 Gy (no deviations). Radiotherapy techniques in this period evolved from 3D-conformal to IMRT and VMAT. In 23 patients electron beams were used.

**Results:**

Median age was 63 years (range 41–88), the majority of patients were female (60%). Twenty three patients were > 75 years old. The TNM stages were I, II, IIIA, and IIIB in 18%, 40%, 15% and 27%, 58% of patients had N0 status. Concurrent mitomycin C and 5-fluoruracil-based chemotherapy was given in 63 patients (64%). Five-year overall survival (OS), disease free survival (DFS), locoregional control (LRC) and colostomy free survival (CFS) were 71%, 80%, 82%, and 82% for the whole group. Results in patients > 75 years of age were not statistically different from those in younger patients. With the introduction of more conformal techniques DFS did not change and toxicities decreased.

**Conclusion:**

Real word treatment outcomes per disease stage were in line with what is reported in literature. Older patients should also be offered treatment with curative intent.

**Supplementary Information:**

The online version contains supplementary material available at 10.1186/s13014-022-02049-8.

## Introduction

Squamous-cell carcinoma of the anus (SCCA) is one of the rarer forms of cancer of the digestive tract, with only 330 new cases in the Netherlands in 2020, an incidence rate of 1.9 per 100.000 people [1]. Sphincter sparing treatment consists of radiotherapy alone in early-stage disease (T1N0 and small T2N0) and radiotherapy with concurrent chemotherapy, typically mitomycin C (MMC) and 5-fluorouracil (5-FU)-based, is given to patients with SCCA at higher stages (larger T2 disease and/or N +) [2–5]. This results in 5 year overall survival rates ranging from 60 to 85 percent and local control rates of 61–83 percent in both prospective studies and retrospective analyses [1, 2, 6, 7]. Acute and long term toxicities are common but reported to be lower with modern radiotherapy techniques VMAT/IMRT versus conventional 3D techniques, thereby also reducing the necessity for therapy breaks [8–12]. In case of residual or recurrent disease an abdominoperineal resection (APR) may be used as salvage treatment in the absence of distant metastases [7]. Because of the rarity of the disease randomized trials are difficult to perform. Older patients are frequently excluded in clinical trials but represent up to 25% of the anal cancer patient population. To gain insight into our institutional results in all age groups and to evaluate trends in outcome and toxicities over time where both radiotherapy techniques and chemotherapy regimens changed a retrospective analysis was performed.

## Materials and methods

### Patients and work up

Medical records from consecutively treated patients treated between 1-1-2009 and 31-12-2018 at our institution were collected with follow up as recorded until 31-12-2020. Our institution is a regional center for treatment of anal cancer patients. Those treated with palliative intent or those who had histologically proven adenocarcinoma were excluded (n = 1 and n = 5 respectively). Staging was done using Magnetic Resonance Imaging (MRI) in 87% of cases, Computed Tomography (CT) in 55%, Positron Emission Tomography (PET) in 22%, conventional chest X-ray in 34%, and/or on indication an ultrasound of the inguinal region in 50% with cytology if possible. Histological biopsies were assessed and graded by the pathology departments of the referring medical centers. Disease staging was done following the American Joint Committee on Cancer 7th edition [13]. In case of debilitating incontinence, in three patients a temporary colostomy was given prior to start of treatment to reinstall continence and increase the possibility of succeeding the chemoradiotherapy treatment. The protocol for this study was approved by Medical Ethics Committee of the Leiden University Medical Center (number G20.026).

### Radiotherapy

Twenty six patients with superficial T1-T2 disease < 4 cm limited to the peri-anal region or anal canal and N0 status received only radiotherapy at 60 Gy in 30 fractions of 2 Gy, usually with a direct electron beam. CTV margin around the tumor was 1 cm, in these patient no elective nodal regions were treated, given dose was the prescribed dose in all patients. This is a conservative approach with limited data to support this practice [5] but with a long history in our institution with good results. Patients with more advanced disease (N + and/or tumor size > 4 cm or small T2 tumors that were not superficially growing) were treated with concurrent chemoradiotherapy, standard dose to the elective nodal regions (perirectal, inguinal, external and internal iliac, obturator and pudendal nodes) was 49.5 Gy in 33 fractions of 1.5 Gy, with a simultaneous integrated boost to macroscopic disease to a total dose of 59.4 Gy in 33 fractions of 1.8 Gy. In case of a sequential boost patients were treated with 25 × 1.8 Gy on the tumor and elective nodal regions followed by a boost on the macroscopic disease, 8 fractions of 1.8 Gy to a total dose of 59.4 Gy. In most patients this was the given dose, six patients were treated with higher doses ranging from 62.2 to 70 Gy. Initially the technique used was 3D conformal radiotherapy (3D), which in 2014 was replaced for IMRT, which further amended to VMAT medio 2016. No treatment gaps were used throughout the time period.

### Chemotherapy

Concurrent chemoradiotherapy consisted of MMC and 5-FU-based chemotherapy. 5-FU has been given as 1000 mg/m^2^ on days 1–4 and 29–32 of radiotherapy. MMC has been given 10 mg/m^2^ on day 1 and 29 [14]. In case of advanced age and/or severe comorbidity MMC was not given. From 2017 onwards capecitabine was given instead of 5-FU, twice daily at 825 mg/m^2^, starting day 1 and to continue until day 33 on radiation days only. From that time onward MMC was only given on day one at a dose of 12 mg/m^2^. Severe toxicity of 5-FU is often the result of deficient function of dihydropyrimidine dehydrogenase caused by genetic polymorphisms in DPYD. Therefore from May 2013 pre-emptive screening for DPYD genotype variants and dose reductions of 5-FU based chemotherapy in heterozygous DPYD variant allele carriers is standard of care [15, 16].

### Follow-up

During treatment patients were seen weekly by the radiation oncologist and nurses to document and treat acute toxicity. In the first-year of follow-up patients were seen frequently until recovery of acute side effects and to evaluate response to treatment, usually at 6 weeks. Then every subsequent 2 months for the remainder of year one. In the second-year follow-up visits were scheduled every 3 months, in the third-year every 4 months, and for the fourth and fifth year every 6 months. No further follow-up was scheduled after 5 years. Imaging with MRI to document treatment response was routinely performed 3 months after completion of radiotherapy. In case of an inconclusive result (a near complete but not total remission, expected to resolve in time) imaging was repeated 8 to 10 weeks later. Further imaging with MRI, PET/CT or other imaging modalities was only performed on suspicion of either metastatic disease or a local recurrence. On indication, depending on complaints and needs of patients, e.g. pain or stool complaints, sexual disfunction or disease-related emotional instability, more or less visits could be scheduled. Treatment response and toxicity were determined and evaluated during these visits. Both relapses and metastatic disease, if present, were confirmed histologically.

### Data collected

The following data were collected from the records: Age at diagnosis, sex, pre-treatment work-up (physical examination and medical imaging) HIV status, tumor histology and grade, maximum tumor size (defined as the greatest dimension), tumor location (categorized as peri-anal, intra-anal, rectal or a combination of these), lymph node involvement, size of the largest pathological lymph node (defined by its greatest dimension), HPV status (either immunohistochemistry or PCR technique) and DYPD mutation status.

Radiotherapy technique and dose were retrieved from the dose report and treatment plan of each patient. If applicable, the chemotherapy plan was retrieved. The justifications for any deviations from standard procedure for either radiotherapy or chemotherapy were retrieved from the patient files and correspondence.

Toxicities reported in the patient files were evaluated following the CTCAE version 4.03 [17]. All toxicities during treatment and within the first 6 weeks after treatment were defined as ‘early toxicity’. Toxicity beyond this period, up to a maximum of 5 years follow-up, was defined as ‘late toxicity’. Only the most severely reported grade a patient experienced of a particular toxicity was recorded. Grade 3 and greater toxicities, urgency grade 2, and incontinence grade 2 were categorized separately.

### Statistical analyses and assessment

The first day of radiotherapy was defined as day 0 for calculations on overall survival (OS), disease free survival (DFS), locoregional control (LRC), Colostomy Free Survival (CFS). Endpoints of OS, DFS, LRC, were estimated with the Kaplan–Meier method whereas Cox proportional hazard models were used to determine the associations of age, sex, stage, T-stage, maximal tumor size N-stage and concurrent chemotherapy with OS, DFS, LRC, and CFS. Log-rank tests were applied to study differences related to period of treatment (< 2014 3D-CRT and $\ge$ 2014 IMRT/VMAT) and age (< 75 or ≥ 75 year).

OS was defined as any survival, DFS as survival without relapse or metastatic progression, LRC as survival duration without local or regional relapse. Date of clinical complete remission (cCR) was defined as the date of reporting cCR or the date of the MRI documenting complete remission, whichever came first. Date of local- or regional relapse was defined as the date of first reported discovery or suspected relapse which was subsequently proven with additional radiological imaging and/or biopsy. In patients without cCR the DFS and LRC was set to 0. When the cause of death was unknown the last date the patient was confirmed disease free was the end-point of DFS. CFS was defined as living without colostomy excluding temporary colostomies that had been reversed. A colostomy that was given prior to treatment and that was not reversed by end of follow-up was considered a day 0 treatment failure.

## Results

### Patient and treatment characteristics

A total of 98 patients treated between January 2009 and December 2018 met the inclusion criteria. Primary characteristics of this study population are described in Table 1. All but one patients were tested for HIV, 3 of them were positive. Twenty-six patients had had a diagnostic surgical tumor resection prior to radiotherapy, of which 19 were R1 and 7 were R2 resections. Eighteen of those 26 patients were treated with local radiotherapy only, 30 × 2 Gy, in the other 8 patients either gross residual disease or nodal involvement was shown upon staging, they were treated with chemoradiation.

**Table 1:** Patient, tumor, and treatment characteristics

Age (years)	Median (range)	63 (41–88)
Gender	Male	39 (40%)
	Female	59 (60%)
HIV status	Negative	94 (96%)
	Positive	3 (3%)
	Unknown	1 (1%)
T stage	1/is	19 (19%)
	2	45 (46%)
	3	29 (30%)
	4	5 (5%)
N stage	0	57 (58%)
	1	16 (16%)
	2	16 (16%)
	3	9 (9%)
TNM staging	I	18 (18%)
	II	39 (40%)
	IIIa	15 (15%)
	IIIb	26 (27%)
Maximum tumor size (mm)	Mean (SD, range)	46 (24, 7–140)
Radiotherapy technique	3D, seq. boost	28 (29%)
	3D, electron boost	3 (3%)
	Electron	23 (24%)
	IMRT, seq. boost	3 (3%)
	IMRT, electron boost	1 (1%)
	IMRT, SIB	13 (13%)
	VMAT	27 (28%)
Concurrent chemotherapy	Yes	63 (64%)
	No	35 (36%)

HPV testing was performed in 28% biopsies of which 90% were positive for high risk HPV. Twelve patients had a diagnosis of a second malignancy before (n = 6) or in the follow-up after their treatment for anal carcinoma (n = 6). In all patients radiotherapy was given without treatment breaks. In 6 patients a higher dose than 60 Gy was applied based on a clinical examination of too much residual disease nearing the end of treatment, dose range 62.2–70 Gy. No dose reductions were applied e.g. in case of toxicities. Sixty-three patients (all stage 2 and 3) received concurrent chemotherapy (64%). Twelve of these patients received no MMC, 10 due to age > 75, 1 due to M. Crohn, and 1 due to poor renal function. In 50 patients DYPD mutations were tested, four patients (8%) had a heterozygote DYPD mutation and received a reduction to their 5-FU or capecitabine dose down to 75%, one patient received both no MMC and a reduced 5-FU dose. Three patients should have received chemoradiotherapy based on their tumor stage but were treated with radiotherapy only, one patient (T2N2) refused chemotherapy, one patient (T2N0) was medically unfit and one other patient (T2N0) for unclear reasons. Thirty five patients were treated with radiotherapy only. This includes the three patients mentioned above, the other patients had disease stages T1N0 (n = 18) or superficial T2N0 with tumor dimensions < 4 cm (n = 14).

During treatment, because of treatment related toxicity, 7 patients received adjustments to their chemotherapy: 5 a dose-reduction, in 2 chemotherapy was prematurely stopped.

### Treatment outcomes

**Fig. 1:**
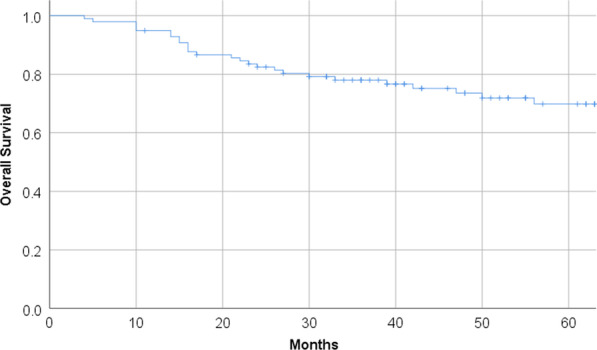
Estimated overall survival for all patients

**Fig. 2:**
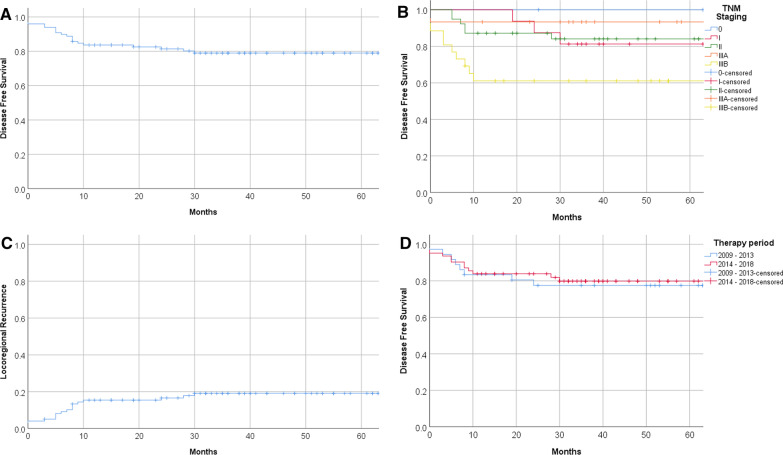
Disease-free survival. **A** Disease-free survival for the whole group. **B** Disease free survival by disease-stage according to AJCC 7th edition; **C** locoregional recurrences for the whole group; **D** disease-free survival of patients with treatment start in 2009–2013 versus 2014–2018

Median follow-up for the cohort was 43 months (range 4–133). Estimated overall survival at 3 and 5 years were 79% and 71% (Fig. 1). An unrelated second malignancy was the cause of death for 4 patients. Estimated DFS at 3 years was 80% (Fig. 2A) with 4 (4%) upfront failures and 16 (16%) relapses after complete remission. Three of the four patients with residual disease after treatment were salvaged with an APR (see Additional file 1: Table S3), the fourth patient experienced a deteriorating health leading to his death without a clear cause of death. The latest relapse in our series occurred at 30 months, with 18 (90%) within the first 24 months after treatment. LRC was achieved in 80 (82%) of patients at 3 years, with a total of 18 locoregional relapses (Fig. 2C). No new locoregional relapses were observed past 30 months. In the 26 patients with T1 and small superficial T2 tumors that were treated with 60 Gy in 30 fractions to the tumor without elective nodal irradiation no isolated regional recurrences occurred. Two regional recurrences occurred simultaneously with distant metastases.

Twenty-seven patients had an anal margin tumor (see Additional file 2: Table S4) of whom 21 were treated with 30 × 2 Gy to the tumor only and 6 received chemoradiation with also treatment on elective nodal regions. Four of these patients experienced a local relapse, 3 in T1/2N0 locally treated patients and one in a patient with T3N2 disease treated with chemoradiation.

By the end of their follow-up 18 patients had a colostomy, of these 13 were due to salvage surgery after relapse, 2 for quality of life due to severe incontinence or urgency, and 3 for other reasons (see Additional file 1: Table S3 for details). Four patients had had a colostomy prior to treatment, of which 3 still had a colostomy at end of follow-up. Five patients with a locoregional relapse did not receive salvage surgery, 3 were inoperable due to simultaneous metastatic progression, 1 due to poor health, and 1 refused further treatment after relapse.

### Univariate analyses

Disease stage, N-status and tumor size were associated with a lower OS (Table 2). Disease stage and N-status were also associated with a lower DFS (Fig. 2B), tumor size however was not. Similarly, a worse LRC was also associated with disease stage and N-status. The CFS however was significantly associated with N-status. Concurrent chemotherapy was not significantly associated with either OS, DFS, LRC or CFS.

**Table 2:** Prognostic factors of survival in univariate analyses

	HR (95% CI)	P value
Overall survival		
Disease stage	1.45 (1.01–2.06)	0.041
T stage	1.36 (0.87–2.13)	0.184
N stage	1.48 (1.06–2.05)	0.021
Max. tumor size	1.01 (1.00–1.03)	0.031
Concurrent chemotherapy	1.50 (0.66–3.42)	0.330
Age	1.02 (0.99–1.06)	0.217
Sex	1.27 (0.60–2.70)	0.532
Disease-free survival		
Disease stage	1.56 (1.03–2.38)	0.037
T stage	1.05 (0.64–1.78)	0.868
N stage	1.66 (1.14–2.42)	0.009
Max. tumor size	1.00 (0.98–1.02)	0.937
Concurrent chemotherapy	1.17 (0.47–2.93)	0.739
Age	1.01 (0.97–1.06)	0.509
Sex	1.66 (0.69–3.98)	0.259
Locoregional control		
Disease stage	1.62 (1.04–2.53)	0.033
T stage	1.17 (0.67–2.03)	0.586
N stage	1.68 (1.13–2.51)	0.010
Max. tumor size	1.00 (0.98–1.02)	0.725
Concurrent chemotherapy	1.26 (0.47–3.35)	0.650
Age	1.01 (0.97–1.06)	0.608
Sex	1.67 (0.66–4.20)	0.278
Colostomy free survival		
Disease stage	1.48 (0.95–2.30)	0.082
T stage	1.30 (0.74–2.30)	0.358
N stage	1.62 (1.07–2.46)	0.022
Max. tumor size	1.01 (0.99–1.03)	0.227
Concurrent chemotherapy	1.26 (0.47–3.36)	0.644
Age	0.99 (0.95–1.03)	0.533
Sex	1.04 (0.40–2.69)	0.931

*HR* hazard ratio, *CI* confidence interval

To analyze time trends as techniques changed, 40 patients treated prior to 2014 (n = 31 with 3D conformal radiotherapy, n = 9 with electrons) were compared to those 58 patients who started after 2014 (n = 44 with IMRT or VMAT and n = 14 with electrons, Fig. 2D). Disease stages were comparable in both groups (data not shown). The group treated before 2014 had an estimated DFS at five years of 78% compared to 81% in the group treated from 2014 onwards. Log-rank difference between these groups was 0.763.

**Fig. 3:**
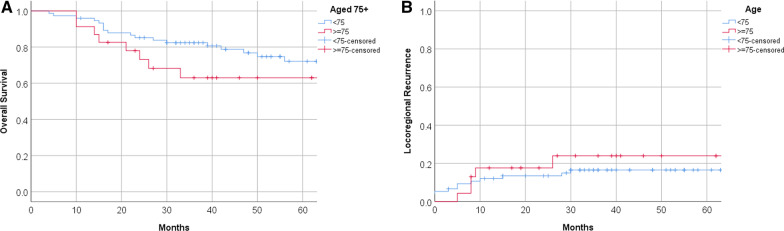
Results in older patients. **A** Estimated overall survival of patients ≥ 75 years old versus patients aged < 75 years; **B** locoregional recurrences in patients ≥ 75 years old versus patients aged < 75

Twenty three patients (23%) were aged 75 years or higher. Ten received radiotherapy only, nine with T1N0 or T2N0 stage disease and one with tumor stage T2N2, this patient refused concurrent chemotherapy. Thirteen patients received chemoradiotherapy for higher stage disease. MMC was frequently omitted as previously described. The estimated 5-year OS in the older age group was lower (60.8% vs. 74.6% in the < 75 group) and the locoregional recurrence was higher (26.1% vs. 18.7% in the < 75 group, Fig. 3A, B). The log-rank test for OS was 0.136, and for the locoregional recurrence 0.496.

### Toxicities

Reported early clinical toxicity of any grade consisted of dermatitis (100% of patients), pain (98%), diarrhea (52%), nausea (35%), urgency (31%), malaise (29%), edema (18%), incontinence for stools (18%), vomiting (11%), and acute cystitis (3%). Leukopenia occurred in 7% of patients treated with concurrent CRT, anemia in 4% and neutropenia in 3%, two patients experienced febrile neutropenia. Early toxicities grade 3 or higher consisted of grade 3 pain in 27% and grade 3or 4 dermatitis in 22% of patients. Other severe toxicities were diarrhea grade 3 or 4 in 5%, grade 3 nausea in 1%, grade 3 vomiting 1% and grade 2 urgency in 8%. Three patients (3%) had such severe anemia they required a transfusion (grade 4).

Reported late toxicities of any grade were pain in 75% of patients, urgency in 53%, fecal incontinence in 49%, diarrhea in 38%, dermatitis in 37%, edema in 26% and nausea in 14%. Higher grade toxicities were 16% grade 2 urgency, 11% grade 3 pain, 3% ≥ grade 3 incontinence, 3% grade 3 dermatitis, and 1 patient with grade 3 diarrhea. Late fecal incontinence in the 27 patients with an anal margin tumor was 33% with 1 patient (4%) with grade 3 incontinence. This is comparable to that in the whole group. Pelvic insufficiency fractures were diagnosed in 3 patients of which 2 were painful and self-limiting. No grade 5 early or late toxicity occurred.

Toxicities changed with the transition of 3D to IMRT/VMAT with an increase in reported acute grade 3 diarrhea from 2.8 to 6.5% and grade 2 urgency from 2.8 to 11.3%, with other toxicities remaining fairly consistent between both techniques. Reported late toxicity differences between patients treated with either 3D or IMRT/VMAT showed an increase in incontinence grade 1–2 from 27.8 to 56.5% but a decrease in reported incontinence grade 3 from 5.6 to 1.6%. Urgency grade 1 rose from 30.6 to 40.3%, but grade 2 reports dropped from 27.8 to 9.7%.

## Discussion

In this study results of our single institution retrospective analysis of 98 consecutively treated patient with SCCA treated in 10 years’ time are described.

The reported 3-and 5 year OS of 78.6% and 71.4% are comparable to that reported in both randomized and other retrospective studies [6, 7, 18, 19]. The ACT I trial reported a 3-year OS of 65% in their CRT group consisting of 292 patients clinically staged as T2N0 or higher [18]. In the Accord 03, a 5-year OS of 71–74.5% was reported in 307 patients with tumors > 4 cm and/or N + disease treated with CRT [19]. The Montpellier study found a 5-year OS of 74% in 193 patients treated with CRT, and Mitra et al. reported a 4-year OS of 85.8% in 99 patients in all disease stages, treated with CRT [6, 7]. The relatively low OS in the ACT I may be attributed to the inclusion of only more advanced stages of disease as well as stage migration over the years (inclusion ended in 1994, MRI and PET CT scans were not widely available at that time). The 4-year OS reported by Mitra et al. is higher than the 3-year OS reported in our study. Although the study populations, treatment period, median age, disease stage distributions are all comparable, this difference might be attributable to chance and limited numbers.

Our reported DFS of 79.6% and LRC of 81.6% at 3 and 5-years compares favorably to the 3-year DFS of the ACT I of 61% and the 5-year DFS of the Montpellier study of 68% [6, 18]. This is likely attributable to inclusion of higher stages and lack of imaging in the ACT I study. The Accord 03 reported an overall relapse free rate of 71.4% and a LRC of 72–87.6% [19]. Mitra et al. reported 83.5% patients were disease-free at the end of follow-up [7]. As with OS however we must again consider that the ACT I trial included more advanced disease stages, and is relatively old, likely attributing to a worse DFS. Compared to the Accord 03, the Montpellier study, and Mitra et al. our DFS is better. The Accord 03 included more advanced disease stages which might negatively affect DFS and LRC and it excluded patients with comorbidities and aged 80 + which should have positively affected outcomes. In the present series 90% of relapses occurred within the first 24 months with the latest relapse at 30 months after treatment. Currently, patients receive a standard of 5-years of follow-up. Considering that in 10 years of curative anal carcinoma treatment all relapses have occurred within three years perhaps a shorter follow-up length is to be discussed with patients [20, 21].

CFS in our study was as high as 81.6%. Of the 18 patients with a colostomy 11 were operated as salvage surgery for relapsed disease. these results are like those reported in the other studies. Where the ACT I reported a CFS of 76.4% in their CRT group, the Accord 03 reported CFS of 69.6–82.4%, the Montpellier study reported 66% CFS, and Mitra et al. reported the highest CFS at 85% [6, 7, 18, 19]. In the ACT I patients only received elective RT and were evaluated after 6 weeks, those with < 50% tumor response were considered for salvage. This practice most likely attributed to a higher rate of colostomies.

Concerns were raised when the RT technique was changed from 3D to more conformal IMRT and VMAT techniques regarding potential regional misses and increased numbers of recurrences. Analyzing the time trend however did not show any significant differences in DFS in our study (78% vs. 81% before and after 2014), further supporting that technique does not affect the primary outcome. Changing the technique from 3D to IMRT/VMAT caused an increase in early reported grade 3 diarrhea and grade 2 urgency. Part of the increase in diarrhea may be attributable to the change from iv 5-FU week 1 and five to oral capecitabine twice daily for the whole treatment period. Severe late incontinence grade 3 dropped from 5.6 to 1.6% with an increase in reported incontinence grade 1–2 from 27.8 to 56.5%. The increase in reported toxicity both early and late is likely also attributable to changes in reporting practices in our clinic, with the advent of the electronic patient file in 2011 and the addition of required toxicity forms since the start of 2018. The decrease in severe toxic effects was the expected effect of switching to IMRT techniques [9, 11, 12].

A significant portion (23%) of our patients were aged 75 and over. Tumor stage did not significantly differ from the under 75 group. Since metastatic disease occurs relatively late in the disease course and the primary tumor can give considerable complaints adequate local treatment is warranted, also in older patients. Due to older patients frequently being excluded from studies, results and outcomes within the geriatric population are often not as clear. For this reason, we analyzed the OS and LRC between patients aged 75 years and older, and those younger than 75. The portion of older patients in our study (23%) is comparable to the portion of older patients with the diagnosis of anal cancer in the Netherlands which is reported to be 28.5% in 2020 [1]. This suggests that older patients are indeed also referred for treatment. Only one patient refused the chemotherapy part of the treatment and frequently MMC was omitted. Older patients had a 5 year OS of 60.8% compared to 74.6% in younger patients, however this was not statistically significant, nor was there a significant difference in LRC. Although a lack of significant difference in OS is unexpected [22], this outcome is most probably due to a to small sample size.

Our study has several limitations. It is a single center retrospective analysis and included patients treated with CRT and with radiotherapy alone. Also the necessity to interpret patient-doctor communication to a toxicity grade is a limitation although reporting in the patient files was quite elaborate. Although radiation techniques changed over time the dose prescribed remained the same. All patients were treated by a team of 2 radiation oncologists and 1 medical oncologist with their residents guarantying consistent treatment choices, techniques and reporting of toxicities.

Whether or not intensification of treatment is useful to improve outcomes remains to be investigated. The Accord 03 study demonstrated that intensifying treatment with either induction chemotherapy or a higher radiotherapy dose did not further improve outcomes [19] whereas the Mitra et al. study reported outcomes comparable, if not marginally better, than our findings but did so with a lower radiotherapy dose [7]. Further studies to find the optimum dose of radiotherapy to balance disease outcome with toxicity are warranted [11, 23]. Results from the ongoing PersonaLising Anal cancer radioTherapy dOse trial (PLATO), incorporating the ACT3, ACT4 and ACT5 trials are eagerly awaited.

## Conclusions

Our 10 year cohort study shows that patients treated with curative intent for anal cancer in our regional referral center achieve OS and DFS comparable to that reported in the literature. With the introduction of more conformal radiation techniques DFS remained similar with lower toxicities. Older patients can be safely treated with slightly adjusted CRT with good results.

## Supplementary Information

**Additional file 1. Table S3**: Details of patients with a colostomy at the end of follow-up.

**Additional file 2. Table S4**: Distribution and treatment of patients according to site of disease.

## Data Availability

The datasets used and/or analysed during the current study are available from the corresponding author on reasonable request.
